# Supplementary material for: Comparative multiomics analysis of cell physiological state after culture in a basket bioreactor
Source: Sci Rep. 2022 Nov 23;12:20161. doi: 10.1038/s41598-022-24687-4 (PMC9686226; doi:10.1038/s41598-022-24687-4)

3666069.1:12855175:12855261:+@NW_023666069.1:12863738:12866576:+@NW_023666069.1:12867948:1286

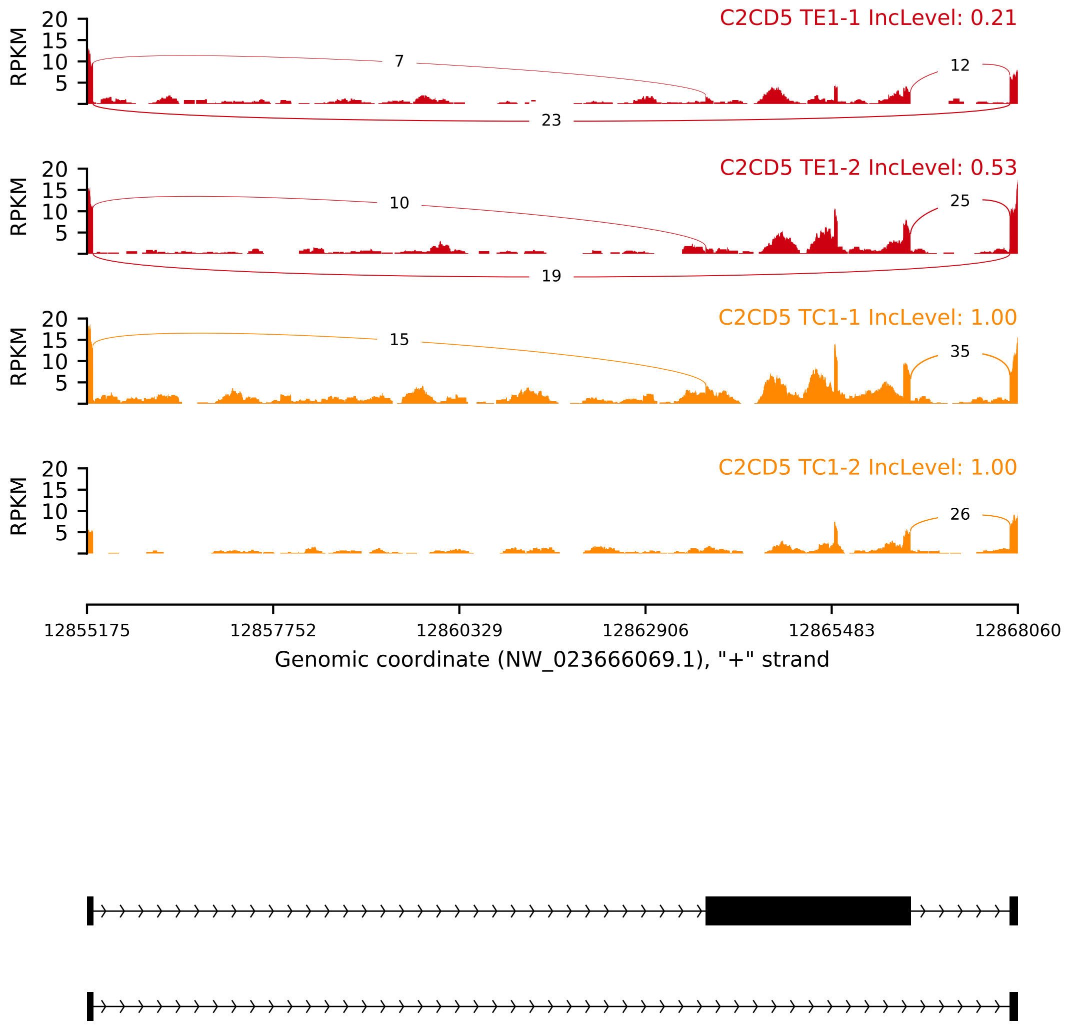

Supplement: Supplementary file 1 — Supplementary Information 1. [file 41598_2022_24687_MOESM1_ESM.zip › raw data/Transcriptomic raw data/7.AS/2.dasplot/TE1vsTC1/SE/13_C2CD5_NW_023666069.1_12855175_12855261_+@NW_023666069.1_12863738_12866576_+@NW_023666069.1_12867948_12868061_+.pdf]
